# Rat Stem-Cell Factor Induces Splenocytes Capable Of Regenerating The Thymus

**DOI:** 10.1155/1992/78751

**Published:** 1992

**Authors:** Eugene S. Medlock, Russell T. Migita, Lisa D. Trebasky, Jerry M. Housman, Gary S. Elliott, R. Wayne Hendren, Randolph B. Deprince, Dale L. Greiner

**Affiliations:** 1 Department of Experimental Hematology Amgen Inc. Amgen Center 1840 Dehavilland Dr. Thousand Oaks California 91320 USA; 2 Department of Pharmaceutics and Drug Delivery Amgen Inc. Amgen Center 1840 Dehavilland Dr. Thousand Oaks California 91320 USA; 3 University of Massachusetts Medical Center Worcester Massachusetts 01605 USA

**Keywords:** Stem-cell factor, thymocytopoiesi, thymus, c-*kit*ligand, cytokines

## Abstract

Cytokine regulation of prethymic T-lymphoid progenitor-cell proliferation and/or differentiation has not been well-defined, although much is known of cytokine regulation of hemopoietic stem- and progenitor-cell development. Here we use a recently identified hemopoietic growth factor, stem-cell factor (SCF) (a form of the c-*kit* ligand), and a transplant model of thymocyte regeneration to assess the effect of SCF on the *in vivo* generation of prethymic, thymocyte progenitor-cell activity. We show that recombinant rat SCF (rrSCF^164^ administered to weanling rats selectively induces an increase in thymocyte progenitor activity in the spleens of treated rats as compared to rats treated with vehicle, polyethylene glycol (PEG)-conjugated rat albumin, or recombinant human granulocyte colony-stimulating factor (rhG-CSF). These data demonstrate that administration of SCF *in vivo* affects extrathymic-origin thymocyte regenerating cells and may influence, directly or indirectly, early prethymic stages of T-cell lymphopoiesis in addition to its known effect on early stages of myelopoiesis and erythropoiesis.


*Developmental Immunology*, 1992, Vol. 3, pp. 35–44
Reprints available directly from the publisher
Photocopying permitted by license only

© 1992 Harwood Academic Publishers GmbH
Printed in the United States of America

### Rat Stem-Cell Factor Induces Splenocytes Capable of Regenerating the Thymus

EUGENE S. MEDLOCK,*† RUSSELL T. MIGITA,† LISA D. TREBASKY,† JERRY M. HOUSMAN,† GARY S. ELLIOTT,† R. WAYNE HENDREN,‡ RANDOLPH B. DEPRINCE,‡ and DALE L. GREINER§

*†Departments of Experimental Hematology and ‡Pharmaceutics and Drug Delivery, Amgen Inc., Amgen Center, 1840 Dehavilland Dr., Thousand Oaks, California 91320*
*§University of Massachusetts Medical Center, Worcester, Massachusetts 01605*

Cytokine regulation of prethymic T-lymphoid progenitor-cell proliferation and/or differentiation has not been well-defined, although much is known of cytokine regulation of hemopoietic stem- and progenitor-cell development. Here we use a recently identified hemopoietic growth factor, stem-cell factor (SCF) (a form of the c-*kit* ligand), and a transplant model of thymocyte regeneration to assess the effect of SCF on the *in vivo* generation of prethymic, thymocyte progenitor-cell activity. We show that recombinant rat SCF ($rrSCF^{164}$) administered to weanling rats selectively induces an increase in thymocyte progenitor activity in the spleens of treated rats as compared to rats treated with vehicle, polyethylene glycol (PEG)-conjugated rat albumin, or recombinant human granulocyte colony-stimulating factor (rhG-CSF). These data demonstrate that administration of SCF *in vivo* affects extrathymic-origin thymocyte regenerating cells and may influence, directly or indirectly, early prethymic stages of T-cell lymphopoiesis in addition to its known effect on early stages of myelopoiesis and erythropoiesis.

KEYWORDS: Stem-cell factor, thymocytopoiesis, thymus, c-*kit* ligand, cytokines.

### INTRODUCTION

Hemopoietic stem and progenitor cells play a central role in the development of all blood-cell lineages (Spangrude et al., 1991). During the past 10 years, much has been discerned concerning the mechanisms by which hemopoietic stem- and progenitor-cell development is regulated, particularly regarding the regulatory roles of cytokines in myeloid lineage development (Clark and Kamen, 1987). Less information has been forthcoming about the possible role(s) of cytokines in regulation of lymphoid stem- or progenitor-cell development (Palacios and Pelkonen, 1988). Recently, a glycoprotein was isolated and identified as a ligand for the tyrosine-kinase receptor c-*kit* and has been variously termed c-*kit*-ligand (Huang et al., 1990), mast cell-growth factor (Williams et al., 1990), stem-cell factor (Zsebo et al., 1990), and steel factor (Matsui et al., 1991). The gene for the c-*kit* ligand has been cloned and the recombinant protein expressed in soluble form (Anderson et al., 1990; Huang et al., 1990; Martin et al., 1990; Langley et al., 1992).

Numerous *in vitro* studies have documented that $rrSCF^{164}$, one form of the c-*kit* ligand, affects the development of a multitude of myelopoietic (Andrews et al., 1990; Migliaccio et al., 1991), erythropoietic (McNiece et al., 1991a), and certain B-lymphoid progenitor cells (McNiece et al., 1991b; Billips et al., 1992). Moreover, recent studies have shown that SCF administered *in vivo* causes an increase in spleen- and marrow-derived spleen colony-forming units (CFU-S) in Steel-Dickie ($Sl/Sl^d$) anemic mice (Bodine et al., 1992). However, little is known of the effect that *in vivo* administration of SCF may have on lymphopoiesis, particularly its possible effect on early T-cell progenitor development.

In the present study, we have administered $rrSCF^{164}$ to rats and evaluated its capability to influence the development of cells able to repopulate the thymus in an irradiation-depen-

*Corresponding author.

dent model of thymocyte regeneration (Greiner et al., 1984). We document that rrSCF[164] causes an increase in spleen cells that can repopulate the thymus of adoptive recipients. These results demonstrate that SCF can affect the development of extrathymic-origin stem/progenitor cells able to reconstitute the thymus in addition to its known effect on myeloid (Andrews et al., 1990; Migliaccio et al., 1991), erythroid (McNiece et al., 1991a), and B-lymphoid progenitor cells (McNiece et al., 1991b; Billips et al., 1992).

### RESULTS

In initial experiments, a factor dose (2.5–500 $\mu$ g/kg) and kinetic (days 0–29) analysis of the hematological response of rats to administration of rrSCF[164] were determined. An optimal increase in peripheral blood leukocytes was observed between days 4 and 18 when using a dose of 500 $\mu$g/kg of rrSCF[164] (Fig. 1). Increases in spleen and peripheral blood leukocyte numbers were observed on day 7 of treatment (Table 1). Spleen weight and cellularity increased approximately 1.5- and 1.4-fold, respectively (Table 1). Increases were apparent in all hemopoietic lineages, including granulocytes, lymphocytes (B and T cells), and monocytes (data not shown). Although bone marrow-cell numbers were unaffected by this treatment (Table 1), cytological analysis revealed a distinct myeloid hyperplasia in the marrow as a result of rrSCF[164] treatment (data not shown). No significant changes in thymus cellularity were observed.

These data suggest that *in vivo* administration of SCF causes hematological changes in both the peripheral and central hemopoietic tissues. The underlying mechanism for these hematological changes are not fully understood, but recent evidence has shown that SCF alone or in combination with other hemopoietically active factors causes the *in vitro* proliferation of myeloid and B-lymphoid progenitor cells and the *in vivo* proliferation of certain types of myeloid stem/progenitor cells identified as CFU-S (Andrews et al., 1990; McNiece et al., 1991a, 1991b; Billips et al., 1992; Bodine et al., 1992). Thus, the increase in mature end-stage progeny as a result of SCF treatment *in vivo* may be directly related to an increase in less mature progenitor cells. Based on our results, 500 $\mu$g/kg of rrSCF[164] was administered daily for 7 days in all subsequent experiments.

We designed the next series of experiments to address whether the increase in lymphoid cells (in particular T-lymphoid cells) in the peripheral blood may be associated with an increase in progenitor cells important for the development of the T-lymphocyte lineage. Spleen and bone marrow from rats treated for 7 days with either rrSCF[164] or vehicle were tested for quantitative differences in thymocyte progenitor-cell activity. In this assay system, graded numbers of cells were adoptively transferred into irradiated, histocompatible, RT-7 (a polymorphic determinant on the rat leukocyte common antigen that is pre-

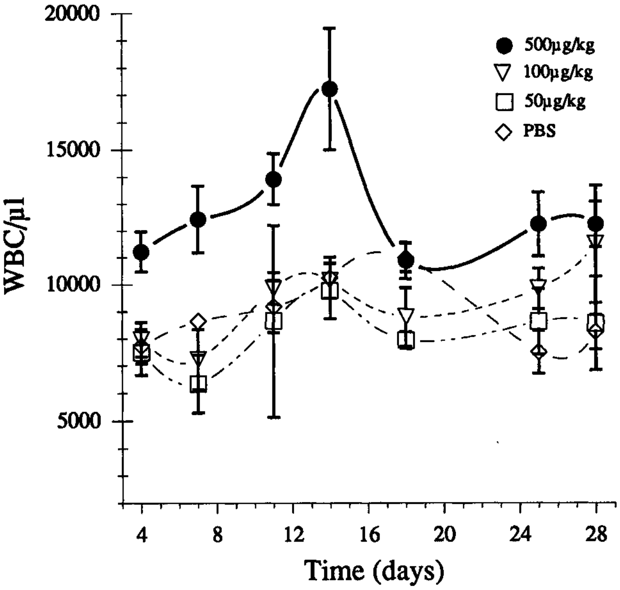

FIGURE 1. White blood cell (WBC) absolute numbers in M520 rats treated with varying dosages of rrSCF[164] for 29 days. Data are presented as the mean±S.D. of three individual rats.

TABLE 1
Cell Number in Lymphohemopoietic Tissues following Stem-Cell Factor Administration[a]

| Treatment | Spleen ($\times10^6$) | Bone marrow ($\times10^6$) | Peripheral blood (cells/$\mu$l$\times10^3$) |
|---|---|---|---|
| Vehicle | 735±45 | 59±5 | 9.6±0.5 |
| rrSCF[164] | 1019±116[b] | 61±20 | 17.7±5.1[b] |
| rhG-CSF | 906±74[b] | 106±7.6[b] | 20.5±4.2[b] |

[a]Each data point represents the mean±standard deviation (S.D.) of eight individual rats from three separate experiments with the exception of the values for rhG-CSF, which are the mean±S.D. of three individual rats. All data were obtained from rats treated for 7 days. Spleen values represent the number of cells per organ and bone marrow values represent the number of cells per femur.
[b]$p<0.01$ versus vehicle-treated values.

sent on all thymocytes) alloantigen disparate recipients (Hale et al., 1987; Sunderland et al., 1979; Thomas et al., 1985). The percentage and number of donor-origin thymocytes, hence thymocyte progenitor activity of the donor inoculum, were determined by flow cytometric analysis of donor alloantigen RT-7$^+$ thymocytes in the recipients. Because the kinetics of thymus regeneration in rats differs among tissue sources of progenitors, it was important to compare several time points of regeneration and transplant cell doses in this model system. For example, the exponential phase of thymus regeneration resulting from a bone marrow transplant occurs between day 12–20 posttransplantation, whereas that due to similar numbers of spleen cells occurs later post-transplantation (Greiner et al., 1984).

Thymus repopulation by bone marrow cells was unaffected by rrSCF$^{164}$-treatment of donors when assayed on either day 18 or 27 after transplantation (Tables 2 and 3, respectively). As shown in Table 2, donor-thymocyte regeneration was proportional to the number of bone marrow cells from vehicle-treated rats transplanted when assayed 18 days posttransplant. No increase or decrease in regeneration was detected when bone marrow cells from rrSCF$^{164}$-treated rats were similarly transplanted. However, as predicted from previous studies (Greiner et al., 1984), thymus regeneration resulting from bone marrow cells was not proportional to transplant cell doses when assayed 27 days posttransplantation (Table 3) (a time during regeneration when donor thymocyte numbers have plateaued). Furthermore, no change in thymocyte regeneration was observed when recipients of bone marrow cells from rrSCF$^{164}$-treated rats were analyzed at day 27 (Table 3).

Recipients of spleen cells from rrSCF$^{164}$-treated rats had greater thymus regeneration than recipients of spleen cells from vehicle-treated rats, but this result was most evident after 27 to 28 days posttransplantation. Recipients of spleen cells from rrSCF$^{164}$-treated rats analyzed on day 18 displayed no significant differences when compared with vehicle-treated rats (Table 2). In contrast, recipients of spleen cells from rrSCF$^{164}$-treated rats analyzed either on day 27, 28, or 29 showed significantly ($p<0.05$) higher regeneration capacity than spleen cells from donors treated similarly with vehicle (Table 3, Table 4, and Fig. 2).

In an additional series of experiments, rats were treated separately with either rat albumin (conjugated with PEG), rrSCF$^{164}$ or rhGCSF for 7 days and spleen cells from each of these donors were assayed for thymocyte progenitor activity. Rats treated with albumin conjugated with PEG were similar to vehicle-treated rats in that no peripheral blood, spleen, or bone marrow hematological changes were observed (data not shown). Furthermore, no change was noted in the ability of splenocytes from PEG-conjugated albumin-treated rats to cause donor-origin thymus regeneration when compared with vehicle-treated spleen cells from several separate experiments

TABLE 2
Thymus Repopulation Capacity of Hemopoietic Tissues from Stem-Cell Factor, or Vehicle-Treated Rats on Day 18[a]

| Treatment | Donor tissue | Cells transferred ($\times 10^7$) | Thymocytes ($\times 10^6$) | Donor-origin thymocytes ($\times 10^6$) |
|---|---|---|---|---|
| Vehicle | Spleen | 1.0 | 387±227 | 7.7±6.9 |
| Vehicle | Spleen | 2.5 | 440±72 | 28.5±14.4 |
| rrSCF$^{164}$ | Spleen | 1.0 | 531±120 | 18.1±5.4[b] |
| rrSCF$^{164}$ | Spleen | 2.5 | 376±101 | 33.3±25.3[b] |
| Vehicle | Bone marrow | 0.5 | 500±195 | 20.6±10.3 |
| Vehicle | Bone marrow | 1.0 | 577±297 | 37.5±17.1 |
| Vehicle | Bone marrow | 2.0 | 518±80 | 115.0±222.0 |
| Vehicle | Bone marrow | 4.0 | 552±89 | 80.9±79.8 |
| rrSCF$^{164}$ | Bone marrow | 0.5 | 611±187 | 27.7±23.6[b] |
| rrSCF$^{164}$ | Bone marrow | 1.0 | 443±89 | 17.3±6.9[b] |
| rrSCF$^{164}$ | Bone marrow | 2.0 | 499±170 | 71.7±13.0[b] |
| rrSCF$^{164}$ | Bone marrow | 4.0 | 584±176 | 96.7±26.4[b] |

[a]Each group represents the mean±standard deviation of four individual recipients analyzed on day 18 posttransplantation.
[b]Not significantly different from vehicle-treated control values.

TABLE 3
Thymus Repopulation Capacity of Hemopoietic Tissues from Stem-Cell Factor, or Vehicle-Treated Rats on Day 27[a]

| Treatment | Donor tissue | Cells transferred (×10⁷) | Thymocytes (×10⁶) | Donor-origin thymocytes (×10⁶) |
|---|---|---|---|---|
| Vehicle | Spleen | 2.5 | 191±73 | 33.2±5.6 |
| Vehicle | Spleen | 5.0 | 200±58 | 69.6±32.2 |
| rrSCF[164] | Spleen | 2.5 | 159±53 | 86.9±12.5[b] |
| rrSCF[164] | Spleen | 5.0 | 238±85 | 170.4±29.6[b] |
| Vehicle | Bone marrow | 1.0 | 199±87 | 136.9±24.8 |
| Vehicle | Bone marrow | 2.5 | 184±21 | 136.8±10.9 |
| Vehicle | Bone marrow | 5.0 | 180±46 | 111.4±34.4 |
| rrSCF[164] | Bone marrow | 1.0 | 222±101 | 130.1±37.6[c] |
| rrSCF[164] | Bone marrow | 2.5 | 204±56 | 117.5±28.8[c] |
| rrSCF[164] | Bone marrow | 5.0 | 178±60 | 113.5±19.5[c] |

[a]Each group represents the mean±standard deviation of 8 to 10 individual recipients analyzed on day 27 posttransplantation.
[b]$p<0.05$ versus vehicle-treated values.
[c]Not significantly different from vehicle-treated control values.

TABLE 4
Thymus Repopulation Capacity of Various Rat Spleen-Cell Populations[a]

| Treatment | Cells transferred (×10⁷) | Thymocytes (×10⁶) | Donor-origin thymocytes (×10⁶) |
|---|---|---|---|
| **Experiment I** | | | |
| Vehicle | 1.0 | 518±53 | 6.3±4.7 |
| Vehicle | 2.5 | 492±103 | 15.9±11.3 |
| Vehicle | 5.0 | 484±86 | 23.0±7.1 |
| rrSCF[164] | 1.0 | 543±132 | 13.6±5.0[c] |
| rrSCF[164] | 2.5 | 490±72 | 46.2±5.4[b] |
| rrSCF[164] | 5.0 | 499±34 | 72.6±3.0[b] |
| Lin⁻ rrSCF[164] | 0.1' | 515±30 | 12.3±2.6 |
| Lin⁻ rrSCF[164] | 0.5 | 536±74 | 32.0±16.0 |
| Lin⁻ rrSCF[164] | 1.0 | 544±79 | 69.0±27.5 |
| **Experiment II** | | | |
| Albumin[PEG] | 1.0 | 746±211 | 12.2±3.2[c] |
| Albumin[PEG] | 2.5 | 709±125 | 12.1±3.3[c] |
| rhG-CSF | 1.0 | 616±100 | 7.3±2.5[c] |
| rhG-CSF | 2.5 | 710±119 | 9.0±5.8[c] |
| rrSCF[164] | 1.0 | 801±153 | 30.0±19.6[d] |
| rrSCF[164] | 2.5 | 774±45 | 55.4±51.7[d] |

[a]Each group represents the mean±standard deviation of four individual recipients analyzed on day 27 in Experiment I and day 28 in Experiment II.
[b]$p<0.005$.
[c]Not significantly different from vehicle-treated control values obtained in two separate experiments.
[d]Although rrSCF[164] regeneration values in Experiment II are not significant, when compared with the recipients of albumin-treated rat splenocytes, or retrospectively compared with vehicle-treated controls, two of four recipients in each cell dose displayed high levels of regeneration.
Thus, in the 1.0×10⁷ cell dose, donor cell levels of 53×10⁶ and 39×10⁶ were seen and in the 2.5×10⁷ cell dose with donor cell levels of 118×10⁶ and 78×10⁶ were seen.

(Table 4). An increase in numbers of spleen, bone marrow, and peripheral blood nucleated cells was observed in rats treated with rhGCSF (Table 1). This was primarily due to an increase in mature polymorphonuclear granulocytes (data not shown). No differences were detected in the ability of spleen cells from rhGCSF-treated rats to cause donor-origin thymus regeneration when compared with splenocytes from rats treated with rat albumin conjugated with PEG (Table 4). Recipients similarly transplanted with splenocytes from rats treated with rrSCF[164] displayed increases in thymocyte regeneration compared with controls confirming our other results (Table 4).

The thymus repopulating cell(s) in the spleen of rrSCF[164]-treated rats was found to be lymphocyte-lineage negative (Lin⁻). Splenocytes of rrSCF[164]-treated rats were depleted of CD4, CD8, and surface immunoglobulin (sIg)-bearing cells with immunomagnetic beads. We recovered approximately 20% of the starting spleen-cell population following the enrichment procedure. When transplanted, the Lin⁻ cells displayed an approximately five-fold enrichment in prothymocyte activity. Thus, $1\times10^7$ Lin⁻ rrSCF[164]-treated splenocytes caused the same level of thymus regeneration that was observed following transplantation of $5\times10^7$ unfractionated rrSCF[164]-treated splenocytes (Table 4). In a separate analysis, the Lin⁻ cell population was found to have ≤10% contaminating Lin⁺ cell types.

Finally, we determined that the increase in spleen-origin thymus repopulating cell activity was a function of increasing doses of rrSCF[164] (Fig. 2). An increase in donor-origin thymocytes of spleen-cell recipients from donors treated with

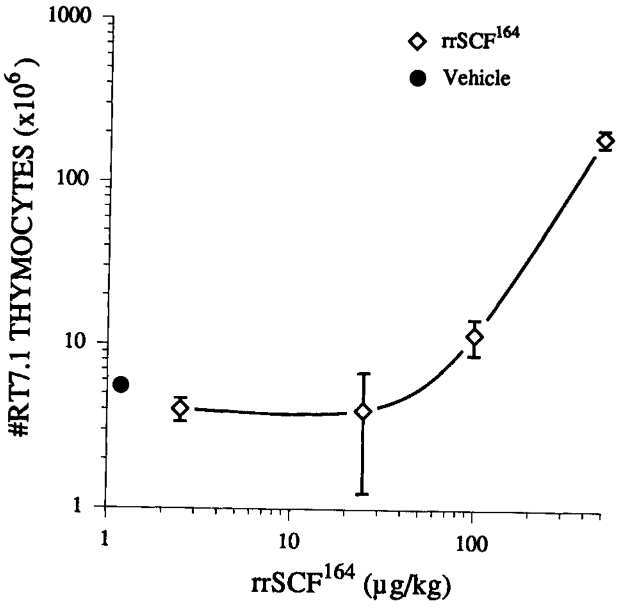

FIGURE 2. Thymocyte regeneration following i.v. adoptive transfer of M520 (RT-7.1) spleen cells ($25\times10^6$/rat) to BUF (RT-7.2) recipients. M520 rats were treated with varying dosages of rrSCF$^{164}$ or vehicle for 7 days at which time the splenocytes were transferred to irradiated BUF recipients. BUF recipients were analyzed on day 29 after transplantation. Data are presented as the mean±S.D. of RT-7.1$^{+}$ (donor-origin) thymocytes from four individual recipients.

100 or 500 $\mu$g/kg of rrSCF$^{164}$ was observed (Fig. 2).

### DISCUSSION

We have used the ability of a cell to generate donor-origin thymocytes in an adoptive recipient as a functional definition of thymocyte progenitor activity. Thymocyte progenitor activity has been detected previously in bone marrow-, spleen-, blood-, and thymus-cell populations, but the factors that regulate the growth and differentiation of prethymic T-lymphocyte progenitor cells are not well-understood (Greiner et al., 1984; Goldschneider et al., 1986; Palacios and Pelkonen, 1988). Furthermore, it is unclear as to whether thymus repopulating activity is due to pluripotent hemopoietic stem cells, a common T- and B-lymphoid stem cell, or a restricted progenitor cell committed only to the T lineage.

We demonstrate in the present study that a population of splenic-origin thymus repopulating cells is induced by *in vivo* treatment with

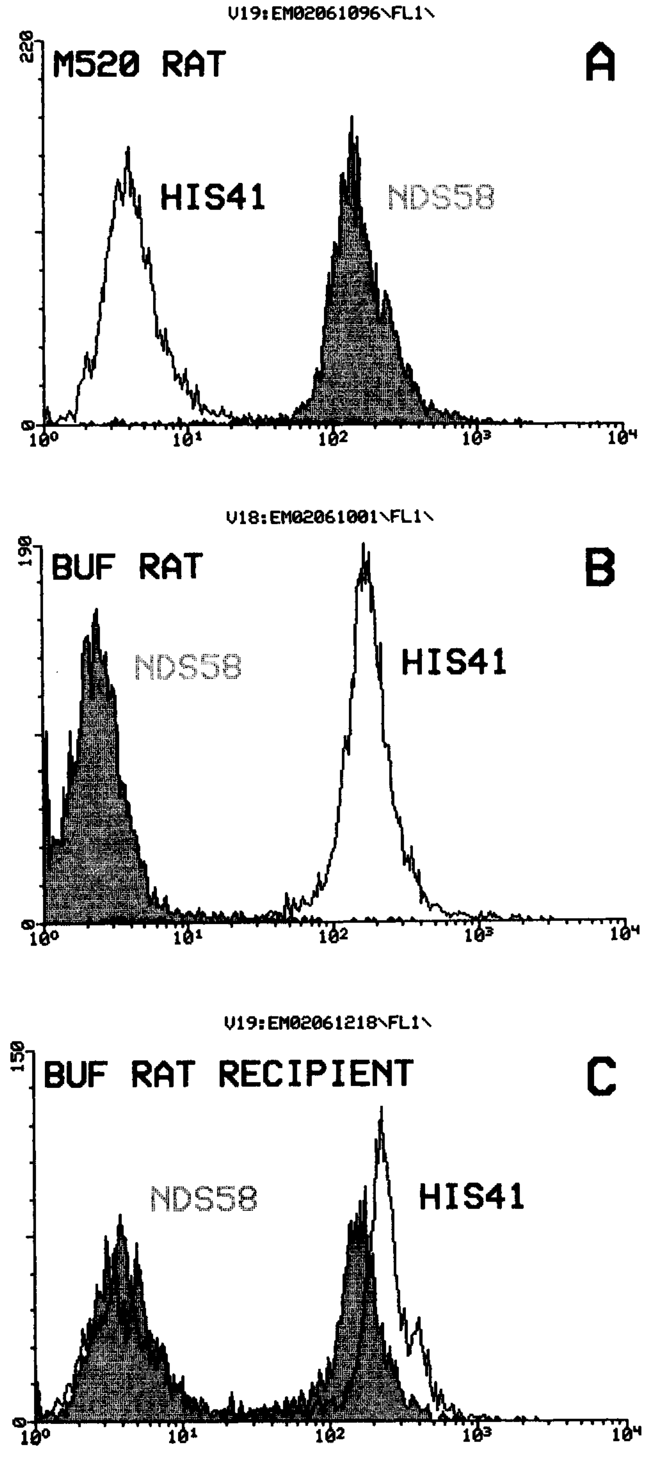

FIGURE 3. Representative fluorescence histogram profiles of NDS58 (RT-7.1) (shaded curve) and HIS41 (RT-7.2) (unshaded curve) reactivity on (A) M520 rat thymocytes, (B) BUF rat thymocytes, and (C) thymocytes from a BUF rat reconstituted 27 days previously with $50\times10^6$ M520 splenocytes. In (C), 49% of the thymocytes were RT-7.1$^{+}$, M520-origin, NDS58 reactive thymocytes and 53% of the thymocytes were RT-7.2$^{+}$, BUF-origin, HIS41 reactive thymocytes.

rrSCF$^{164}$. Thus, we show that spleen-origin thymocyte progenitor activity was increased in rats administered rrSCF$^{164}$ *in vivo* for 7 days. In contrast, bone marrow-origin thymocyte progenitor activity was not increased by similar treatment. Our data indicate that elevated levels of donor-origin thymocytes due to spleen-cell transplants were most evident on days 27–28 posttransplantation. This is in contrast to the kinetics of regeneration by bone marrow progenitors that reach plateau levels of regeneration by day 20 (Greiner et al., 1984). This apparent difference in the kinetics of thymocytopoiesis may be due in part to differences in the relative numbers of thymus repopulating cells in the spleen versus the bone marrow, to differences in thymus colonizing ability of spleen cells versus bone marrow cells, and/or to differences in the developmental stage of spleen versus bone marrow progenitors.

Previous reports have shown that SCF either alone or in combination with other hemopoietic growth factors or interleukins stimulates primitive stem- and progenitor-cell populations important for the production of macrophages, granulocytes, erythrocytes, and B-lymphoic progenitor cells (Andrews et al., 1990; McNiece et al., 1991a, 1991b; Migliaccio et al., 1991; Billips et al., 1992; Bodine et al., 1992). Moreover, SCF has been shown to stimulate the *in vitro* growth of enriched marrow-derived stem-cell populations (Bernstein et al., 1991; de Vries et al., 1991; Williams et al., 1992). Our results would suggest that SCF stimulates the proliferation and/or differentiation of a primitive cell in the spleen that has the capacity of migrating to the thymus and generating thymocytes. It is not clear whether the cell population affected in the spleen is distinguishable from a pluripotential hemopoietic stem cell capable of generating all hemopoietic lineages (Spangrude et al., 1989; Jordan et al., 1990; Spangrude and Scollay, 1990). However, our lineage-depletion studies as well as previously published studies in which mature T-lymphocytes were transplanted indicate that thymus reconstitution was most likely not the result of thymus colonization by nonactivated, mature, peripheral T cells (Goldschneider et al., 1986; Agus et al., 1991).

A more trivial explanation of our results may be that rrSCF$^{164}$ causes shunting of thymus repopulating cells from the marrow to the spleen via the vascular system. Administration of other hemopoietically active cytokines, such as rhG-CSF, has been shown to induce shunting of stem and progenitor cells to the peripheral blood from the bone marrow (Molineux et al., 1990). In our model system, administration of rhG-CSF to rats caused increased levels of granulocytes in peripheral blood, spleen, or bone marrow. However, rhG-CSF did not cause an apparent shunting of thymus repopulating cells to the spleen from the bone marrow because spleen thymocyte progenitor activity levels in these rhG-CSF-treated animals were not different from albumin-treated controls or vehicle-treated controls from other experiments. Additionally, thymocyte progenitor activity levels in the bone marrow of rrSCF$^{164}$ treated rats did not decrease but remained stable following treatment. In preliminary experiments, we have observed that peripheral blood nucleated cells from rrSCF$^{164}$ treated rats did not differ in thymocyte progenitor activity compared with controls (Medlock and Housman, unpublished observations) further suggesting that the increase in thymus repopulating cells in the spleen was not due to shunting of such cells from the bone marrow via the vascular system.

Another, less likely mechanism that might explain our results is that the PEG conjugated to the rrSCF$^{164}$ was the contributing factor in stimulating the increase in spleen-origin thymocyte progenitor activity. Rats treated with 500 $\mu$g/kg of PEG-conjugated rat albumin displayed no change in the levels of thymocyte progenitor activity when compared to vehicle-treated rats, suggesting that PEG itself did not induce splenic thymocyte progenitor activity.

In contrast to the previously mentioned mechanisms, rrSCF$^{164}$ may instead induce the development of a splenic accessory cell that cocolonizes the thymus along with spleen-derived thymus repopulating cells, enhancing their thymocyte regenerating ability. This putative accessory cell, although it has not been physically separated from a thymocyte progenitor cell, appears to be of myeloid, not lymphoid, origin or may derive from hemopoietic stem-cell differentiation in the thymus (Fowlkes and Pardell, 1989; Spangrude and Scollay, 1990). Furthermore, *in vitro* (Deluca, 1986; Deluca and Misel, 1986) and *in vivo* (Greiner et al., 1986; Komschlies et al., 1987; Hayes et al., 1992) thymic repopulation studies have suggested that this accessory cell may be a rate-limiting factor in thymocyte repopulation.

Thus, rrSCF$^{164}$ may affect a myeloid accessory cell, rather than a lymphoid progenitor cell, resulting in increased accessory cell activity and enhanced thymocyte repopulation.

Although thymocyte progenitor activity has been identified in many tissues throughout the body (Goldschneider et al., 1986), the primary source, or at least the source with the largest population, is the bone marrow in the adult (Kadish and Basch, 1986; Greiner et al., 1984; Goldschneider et al., 1986; Katsura et al., 1986). However, we have observed that the primary site of rrSCF$^{164}$ action on this type of cell activity is selective to the spleen, an apparently secondary source of thymocyte progenitor activity in the rat (Greiner et al., 1984; Goldschneider et al., 1986). This apparent discrepancy might be explained by the simple fact that PEG-conjugated rrSCF$^{164}$ was unable to stimulate the bone marrow perhaps as a result of the inability of PEG-conjugated rrSCF$^{164}$ to enter the bone marrow microenvironment. This seems unlikely because we have observed that, although bone marrow cellular numbers do not change as a result of treatment, there is a bone marrow cytological change with elevated levels of morphologically detectable myeloid precursor cells. Alternatively, the increase in spleen thymocyte progenitor activity may indirectly suggest that the bone marrow and spleen thymus repopulating cells might be developmentally distinct types of hemolymphopoietic cells. Thus, rrSCF$^{164}$ may act on one type of thymus repopulating cell but not the other. Abramson et al. (1977) were the first to demonstrate that T-restricted stem progenitor cells may exist in the bone marrow using radiation-induced chromosomal aberrations as a clonal marker. Dick et al. (1985) detected similar cells using insertion of a selectable gene as a clonal marker. Moreover, it is certain that pluripotent hemopoietic stem cells are able to reconstitute all blood lineages including T cells that are generated in the thymus (Jordan et al., 1990). Indeed, certain studies have suggested that prethymic-origin thymus repopulating cells may be separated into two distinct cell phenotypes in the mouse (Spangrude et al., 1989). The first is a Thy1$^{Lo}$, Lin$^{-}$, Sca-1$^{+}$ cell type similar to hemopoietic stem cells, and the second is Thy1$^{Lo}$, Lin$^{+}$, Sca1$^{+}$, which contains no hemopoietic stem-cell activity. However, the demonstration of two separate populations of thymus repopulating cells in rat bone marrow and/or spleen must await cellular purification of thymocyte progenitor cells from each source with subsequent comparison of their respective antigenic and functional phenotypes.

In conclusion, we have detected an additional biological activity of rrSCF$^{164}$ in which thymocyte progenitor activity is selectively altered in the spleens of rrSCF$^{164}$-treated rats. Future studies will be directed toward the isolation and characterization of these spleen-derived thymocyte progenitor cells for the purpose of comparing and contrasting their physical and functional properties with those present in the bone marrow.

### MATERIALS AND METHODS

### Growth Factors

Recombinant rrSCF$^{164}$ and rhG-CSF were purified after expression in *Escherichia coli*, as described previously (Souza et al., 1986; Langley et al., 1992). Methoxypolyethylene glycol (MW=6000) was obtained from Union Carbide Chemicals and Plastic Company (South Charleston, WV) and activated as previously described (Veronese et al., 1989). PEG-conjugated SCF, containing two to three PEG strands per SCF subunit, was prepared by an adaptation of a previously published method (Tanaka et al., 1991) and used because previous studies have shown that PEG improves the stability of other cytokines *in vivo* (Katre et al., 1986; Tanaka et al., 1991). PEG-conjugated rat albumin was prepared similarly.

### rrSCF$^{164}$ Treatment Protocol

In initial experiments, factor dose and kinetic responses of rats to rrSCF$^{164}$ were performed. In most subsequent experiments, 4- to 6-week-old M520 rats (NCI, Frederick, MD) were treated daily with 500 $\mu$g/kg of rrSCF$^{164}$ or vehicle (buffered saline with 1% fetal bovine serum) subcutaneously for 7 days. In certain experiments, M520 rats were treated with either 500 $\mu$g/kg PEG-conjugated albumin or 50 $\mu$g/kg of rhG-CSF. On day 7, blood, spleen, and bone marrow cells were collected and analyzed.

### Cell Suspensions

Peripheral blood was collected using EDTA as an anticoagulant. Bone marrow suspensions were prepared by flushing the femur with medium (RPMI 1640) and spleen-cell suspensions were made by extrusion of the spleens through a 50-mesh wire screen into medium. Cell counts were performed using a Coulter Counter Channelizer (Hialeah, FL). In certain experiments, blood was collected on days 4, 7, 11, 14, 18, 25, and 29 of factor treatment and analyzed by flow cytometry and Coulter analysis. All bone marrow-cell transplants were done by intravenous injection. Thymi from recipients were removed at various times after transplantation and cell suspensions prepared. Absolute nucleated cell numbers were determined by Coulter analysis.

### Irradiation

Male BUF (Harlan) (alloantigen-RT7.2$^+$) rats aged 4–6 weeks were whole body irradiated with 7.5 Gy using a $^{137}$Cs source (Gammacell 40, Atomic Energy of Canada, Ltd.) 4–6 hours prior to transplantation with M520 (alloantigen-RT7.1$^+$) tissues.

### Antibody Labeling and Flow Cytometry Analysis

Each thymus-cell suspension was incubated with antibodies to the RT7.1 alloantigen (clone NDS 58 or BC84) or RT7.2 alloantigen (clone HIS 41, Bioproducts for Science, Indianapolis, IN) and developed for fluorescence analysis with goat anti-rat Ig FITC or goat anti-mouse Ig FITC, respectively. Red blood cells were removed following staining using lysing buffer according to the manufacturer's protocol (Becton Dickinson, Milpitas, CA). Labeled cells were fixed in 1% buffered formalin and analyzed with a FACScan cell analysis system (Becton Dickinson) within 24–48 hr. The percentage of positive cells for each antibody was determined from histogram profiles and the absolute number of donor (RT7.1$^+$) cells was determined by the following formula: No. donor cells=% NDS58$^+$ cells −% rat Ig$^+$ cells× absolute number of thymocytes. As shown, NDS58 labels 100% of M520=origin thymocytes and 0% of BUF-origin thymocytes, and HIS41 labels 100% of BUF-origin thymocytes and 0% of M520-origin thymocytes (Fig. 3). A representative BUF recipient of M520 splenocytes is shown that indicates that NDS58 discriminates approximately 49% of this regenerating thymus (Fig. 3). The data comparing rrSCF$^{164}$ treatment with vehicle treatment in Tables 2, 3, and 4, and Fig. 2 have been reproduced in nine separate experiments. All statistics have been performed using the student's "T" test for grouped data.

### Lin$^-$-Cell Preparation

Lin$^-$ cells were prepared by labeling spleen cells from rrSCF$^{164}$-treated rats with mouse antibodies to rat CD4 (clone W3/25), CD8 (clone OX8), (Accurate Chemical and Scientific Corp., Westbury, NY) and sIg (Southern Biotechnology Associates, Birmingham, AL). Labeled cells were reacted and depleted twice with immunomagnetic beads conjugated with sheep anti-mouse Ig (Dynal Corp., Oslo, Norway). Nonadherent cells were recovered, washed twice, and suspended at the appropriate concentration for i.v., injection. Lin$^-$ cells were assayed for contaminating CD4, CD8, and sIg-bearing cells by staining the Lin$^-$ cells with a FITC-conjugated goat anti-mouse Ig and analyzed on the FACScan.

### NOTE ADDED IN PROOF

In a recent study by Chervenak et al., 1992, J. Immunol. 149: 2851–2856, it has been shown that c-kit ligand (MGF) induces the expansion of a population of thymocyte progenitor cells when cultured *in vitro* with MGF. This *in vitro* evidence confirms and extends our *in vivo* evidence reported herein.

### ACKNOWLEDGMENTS

We thank the staff members of the Amgen Laboratory Animal Care Facility for their patience during these studies, gratefully acknowledge the assistance of Maureen Mullally, and thank Joan Bennett for the preparation of the manuscript.

*(Received May 22, 1992)*

*(Accepted August 31, 1992)*

### REFERENCES

Abramson S., Miller R., and Phillips R. (1977). The identification by H bone marrow of pluripotent and restricted stem cells of the myeloid and lymphoid system. J. Exp. Med. **145:** 1567–1579.

Agus D.B., Surgh C.D., and Sprent J. (1991). Reentry of T cells to the adult thymus is restricted to activated T cells. J. Exp. Med. **173:** 1039–1046.

Anderson D.M., Lyman S.D., Baird A., Wignall J.M., Eisenman J., Rauch C., March C.J., Boswell H.S., Gimpel S.D., Cosman D., and Williams D.E. (1990). Molecular cloning of mast cell growth factor, a hematopoietic factor that is active in both membrane and soluble forms. Cell **65:** 235–243.

Andrews R.G., Knitter G.H., Bartelmez S.H., Langley K.E., Farrar D., Hendren R.W., Appelbaum F., Bernstein I.D., and Zsebo K.M. (1990). Recombinant human stem cell factor, a c-*kit* ligand, stimulates hematopoiesis in primates. Blood **78:** 1975–1980.

Bernstein I.D., Andrews R.G., and Zsebo K.M. (1991). Recombinant human stem cell factor enhances the formation of colonies by $CD34^+$ and $CD34^+$ $Lin^-$ cells, and the generation of colony-forming cell progeny from $CD34^+$ $Lin^-$ cells cultured with interleukin-3, granulocyte-colony stimulating factor, or granulocyte-macrophage colony-stimulating factor. Blood **77:** 2316–2321.

Billips L.G., Petitte P., Dorshkind K., Narayanan R., Chiu C.P., and Landreth K.S. (1992). Differential roles of stromal cells interleukin-7, and *kit*-ligand in the regulation of B lymphopoiesis. Blood **79:** 1185–1192.

Bodine D.M., Orlic D., Birkett N.C., Seidel N.E., and Zsebo K.M. (1992). Stem cell factor increases colony-stimulating unit-spleen number *in vitro* in synergy with interleukin-6, and *in vivo* in $Sl/Sl^d$ mice as a single factor. Blood **79:** 913–919.

Clark S.C., and Kamen R. (1987). The human hematopoietic colony-stimulating factors. Science (Washington, D.C.) **236:** 1229–1237.

Deluca D. (1986). Ia-positive nonlymphoid cells and T cell development in murine fetal thymus organ cultures: Monoclonal anti-Ia antibodies inhibit the development of T cells. J. Immunol. **136:** 430–439.

Deluca D., and Misel S.B. (1986). Ia-positive nonlymphoid cells and T cell development in murine fetal thymus organ cultures: interleukin-1 circumvents the block in T cell differentiation induced by monoclonal anti-Ia antibodies. J. Immunol. **137:** 1435–1441.

de Vries P., Brasel K.A., Eisenman J.R., Alpert A.R., and Williams D.E. (1991). The effect of recombinant mast cell growth factor on purified murine hematopoietic stem cells. J. Exp. Med. **173:** 1205–1211.

Dick J., Magli M., Huszar D., Phillips R., and Bernstein A. (1985). Introduction of a selectable gene into primitive stem cells capable of long-term reconstitution of the hemopoietic system of $W/W^v$ mice. Cell **42:** 71–79.

Fowlkes B.J., and Pardell D.M. (1989). Molecular and cellular events of T cell development. Adv. Immunol. **44:** 207–264.

Goldschneider I., Komschlies K.L., and Greiner D.L. (1986). Studies of thymocytopoiesis in rats and mice. I. Kinetics of appearance of thymocytes using a direct intrathymic adoptive transfer assay for thymocyte precursors. J. Exp. Med. **163:** 1–17.

Greiner D.L., Goldschneider I., Komschlies K.L., Medlock E.S., Bollum F.J., and Schultz L.D. (1986). Defective lymphopoiesis in bone marrow of motheaten *(me/me)* and viable motheaten *($me^v/me^v$)* mutant mice. I. Analysis of development of prothymocytes, early B lineage cells and terminal deoxynucleotidyl transferase positive cells. J. Exp. Med. **164:** 1129–1144.

Greiner D.L., Goldschneider I., and Lubroff D.M. (1984). Identification of thymocyte progenitors in hemopoietic tissues of the rat. I. A quantitative assay system for thymocyte regeneration. Thymus **6:** 181–199.

Hale M.L., Greiner D.L., and McCarthy K.F. (1987). Characterization of rat prothymocyte with monoclonal antibodies recognizing rat lymphocyte membrane antigenic determinants. Cell. Immunol. **107:** 188–200.

Hayes S.M., Shultz L.D., and Grenier D.L. (1992). Thymic involution in viable motheaten *($me^v$)* mice is associated with a loss of intrathymic precursor activity. Dev. Immunol. **2:** 191–205.

Huang E., Nocka K., Beier D.R., Chu T.-Y., Bick J., Lahm H.W., Wellner D., Leder P., and Besmer P. (1990). The hematopoietic growth factor KL is encoded by the Sl locus and is the ligand of the c-*kit* receptor, the gene product of the W locus. Cell **63:** 225–233.

Jordan C.T., McKearn J.P., and Lemischka I.R. (1990). Cellular and developmental properties of fetal hematopoietic stem cells. Cell **61:** 953–963.

Kadish J.L., and Basch R.S. (1976). Hematopoietic thymocyte precursors. I. Assay and kinetics of the appearance of progency. J. Exp. Med. **143:** 1082–1099.

Katre N.V., Knauf M.J., and Laird W.J. (1986). Chemical modification of recombinant interleukin-2 by polyethylene glycol increases its potency in the murine MethA sarcoma model. Proc. Natl. Acad. Sci. USA **84:** 1487–1491.

Katsura Y., Kina T., Amagai T., Tsubata T., Hirayoshi K., Takaoki Y., Sado T., and Nishikawa S.I. (1986). Limit dilution analysis of the stem cells for T cell lineage. J. Immunol. **137:** 2434–2439.

Komschlies K.L., Greiner D.L., Shultz L., and Goldschneider I. (1987). Defective lymphopoiesis in the bone marrow of motheaten *(me/me)* and viable motheaten *($me^v/me^v$)* mutant mice. III. Normal mouse bone marrow cells enable *$me^v/me^v$* prothymocytes to generate thymocytes after intravenous transfer. J. Exp. Med. **166:** 1162–1167.

Langley K.E., Wypych J., Mendiaz E.A., Clogston C.L., Parker V.P., Farrar D.H., Brothers M.O., Satyagal V.N., Leslie I., Birkett N.C., Smith K.A., Baltera R.F., Lyons P.E., Hogan J.M., Crandall C., Boone T.C., Pope J.A., Karkare S.B., Zsebo K.M., Sachdev R.K., and Lu H.S. (1992). Purification and characterization of soluble forms of human and rat stem cell factor recombinantly expressed by *Escherichia coli* and by Chinese hamster ovary cells. Arch. Bioch. Biophys. **295:** 21–28.

McNiece I.K., Langley K.E., and Zsebo K.M. (1991a). Recombinant human stem cell factor synergizes with GM-CSF, G-CSF, IL-3 and EPO to stimulate progenitor cells of the myeloid and erythroid lineages. Exp. Hematol. **19:** 226–231.

McNiece I.K., Langley K.E., and Zsebo K.M. (1991b). The roles of recombinant stem cell factor in early B cell development. Synergistic interaction with IL-7. J. Immunol. **146:** 3785–3790.

Martin F.H., Suggs S.V., Langley K.E., Lu H.S., Ting J., Okino K.H., Morris C.F., McNiece I.K., Jacobsen F.W., Mendiaz E.A., Birkett N.C., Smith K.A., Johnson M.J., Parker V.P., Flores J.C., Patel A.C., Fisher E.F., Erjavec H.O., Herrera C.J., Wypych J., Sachdev R.K., Pope K.A., Leslie I., Wen D., Lin C.W., Cupples R.L., and Zsebo K.M. (1990). Primary structure and functional expression of rat and human stem cell factor cDNA's. Cell **63:** 203–211.

Matsui Y., Toksoz D., Nishikawa S., Nishikawa S.I., Williams D., Zsebo K. and Hogan B.L.M. (1991). Effect of *Steel* factor and leukemia inhibitory factor on murine primordial germ cells in culture. Nature **353:** 750–752.

Migliaccio G., Migliaccio A.R., Druzin M.L., Giardina P.J.V., Zsebo K.M., and Adamson J.W. (1991). Effects of recombinant human stem cell factor (SCF) on the growth of human progenitor cells *in vitro*. J. Cell Physiol. **148:** 503–509.

Molineux G., Pojda Z., Hamson I.N., Lord B.I., and Dexter

T.M. (1990). Transplantation potential of peripheral blood stem cells induced by granulocyte colony-stimulating factor. Blood **76:** 2153–2158.

Palacios R., and Pelkonen J. (1988). Prethymic and intrathymic mouse T cell progenitors: Growth requirements and analysis of the expression of genes encoding TCR/T3 components and other T cell-specific molecules. Immunol. Rev. **104:** 5–27.

Souza L.M., Boone T.C., Gabrilove J., Lai P.H., Zsebo K.M., Murdock D.C., Chazin V.R., Bruszewski J., Lu H., Chen K.K., Barendt J., Platzer E., and Moore M.A.S. (1986). Recombinant human granulocyte colony-stimulating factor: Effects on normal and leukemic myeloid cells. Science **232:** 61–65.

Spangrude G.J., Klein J., Heimfeld S., Aihara Y., and Weissman I.L. (1989). Two monoclonal antibodies identify thymic repopulating cells in mouse bone marrow. J. Immunol. **142:** 425–430.

Spangrude G.J., and Scollay R. (1990). Differentiation of hematopoietic stem cells in irradiated mouse thymic lobes. Kinetics and phenotype of progeny. J. Immunol. **145:** 3661–3668.

Spangrude G.J., Smith L., Uchida N., Ikuta K., Heimfeld S., Friedman J., and Weissman I.L. (1991). Mouse hemopoietic stem cells. Blood **78:** 1395–1402.

Sunderland C.A., McMaster W.R., and Williams A.F. (1979). Purification with monoclonal antibody of a predominant leukocyte-common antigen and glycoprotein from rat thymocytes. Eur. J. Immunol. **9:** 155–159.

Tanaka H., Satake-Ishikawa R., Ishikawa M., Matsuki S., and Asano K. (1991). Pharmacokinetics of recombinant human granulocyte-colony stimulating factor conjugated to polyethylene glycol in rats. Cancer Res. **51:** 3710–3714.

Thomas M.L., Barclay A.N., Gagnon J., and Williams A.F. (1985). Evidence from cDNA clones that the rat leukocyte-common antigen (T200) spans the lipid bilayer and contains a cytoplasmic domain of 80,000 Mr. Cell **41:** 83–93.

Veronese F.M., Caliceti P., Pastorino A., Schiavon O., and Sartore L. (1989). Preparation, physiochemical and pharmacokinetic characterization of monomethoxypoly (ethylene glycol)-derivatized superoxide dismutase. J. Controlled Release **10:** 145–154.

Williams N., Bertoncello I., Kovnoudias H., Zsebo K., and McNiece I. (1992). Recombinant rat stem cell factor stimulates the amplification and differentiation of fractionated mouse stem cell populations. Blood **79:** 58–64.

Williams D.E., Eisenman J., Baird A., Rauch C., van Nes K., March C.J., Park L.S., Martin U., Mochizuki D.Y., Boswell H.S., Burgess G.S., Cosman D., and Lyman S.D. (1990). Identification of a ligand for the c-*kit* protooncogene. Cell **63:** 167–174.

Zsebo K.M., Wypych J., McNiece I.K., Lu H.S., Smith K.A., Karkare S.B., Sachdev R.K., Yuchenkoff V.N., Birkett N.C., Williams L.R., Satyagal V.N., Tung W., Bosselman R.A., Mendiaz E.A., and Langley K.E. (1990). Identification, purification, and biological characterization of hematopoietic stem cell factor from Buffalo rat liver-conditioned medium. Cell **63:** 195–201.

